# Simulation Optimization of AlGaN/GaN SBD with Field Plate Structures and Recessed Anode

**DOI:** 10.3390/mi14061121

**Published:** 2023-05-26

**Authors:** Tao Xu, Ziqi Tang, Ziyou Zhou, Bing Zhou

**Affiliations:** 1 Key Laboratory of New Processing Technology for Nonferrous Metals and Materials of Ministry of Education, School of Materials Science and Engineering, Collaborative Innovation Center for Exploration of Nonferrous Metal Deposits and Efficient Utilization of Resources, Guilin University of Technology, Guilin 541004, China; xutao202303@163.com (T.X.); zz1759581482@163.com (Z.Z.); 2 Ningbo Haitechuang Electric Control Co., Ltd., Ningbo 315000, China; tangziqi2021@126.com

**Keywords:** AlGaN/GaN SBD, recessed anode, TCAD simulation, field plate, FOM

## Abstract

This study investigated several AlGaN/GaN Schottky Barrier Diodes (SBDs) with different designs to achieve device optimization. First, the optimal electrode spacing, etching depth, and field plate size of the devices were measured using Technology Computer-Aided Design (TCAD) software by Silvaco, and analysis of the electrical behavior of the device was based on the simulation results, and several AlGaN/GaN SBD chips were designed and prepared. The experimental results revealed that the recessed anode can increase the forward current and reduce the on-resistance. An etched depth of 30 nm could obtain a turn-on voltage of 0.75 V and a forward current density of 216 mA/mm. A breakdown voltage of 1043 V and a power figure of merit (FOM) value of 572.6 MW/cm^2^ was obtained with a 3 μm field plate. Experiments and simulations confirmed that the recessed anode and field plate structure could increase the breakdown voltage and forward current and improve the FOM value, resulting in higher electrical performance and a wider range of application scenarios.

## 1. Introduction

Schottky barrier diodes (SBDs) are widely used in switching power supplies, inverters, and drivers, which typically require characteristics, such as fast reverse recovery, low on-state voltage drop, and high voltage resistance to reduce power dissipation [1,2,3]. However, Si-based Schottky barrier diodes [4] generally have problems with high on-state turn-on voltages, high reverse bias leakage, and low breakdown voltages due to the material properties. Simultaneously, SiC-based SBDs also limit development due to the high manufacturing costs [5]. GaN-based SBD devices have gained significant interest in high power [6,7] and speed applications due to their excellent material properties, such as high carrier density, electron saturation rate, and breakdown voltage. The large forbidden bandwidth of 3.4 eV allows GaN-based SBDs to have good high-voltage resistance, but this also results in a relatively high on-state voltage (Von), which can increase operating losses and reduce efficiency [8].

Previous studies have shown that the use of recessed anode treatment in AlGaN/GaN SBD devices can reduce the on-state voltage and leakage current of the device. Meanwhile, field plate technology [9] has proven effective in increasing the breakdown voltage of SBDs. In 2019, Xu et al. prepared recessed-anode AlGaN/GaN SBD devices. They investigated the breakdown voltage enhancement by conducting simulations with different field plate lengths and passivation layer thicknesses. The specific on-state resistance (Ron.sp) was 3.7 mΩ·cm^2^, and the breakdown voltage was up to 3.4 KV [10]. Chuan-Wei Tsou et al., in 2016, prepared recessed anode SBD devices and investigated the effect of the surface roughness of the recessed anode on the device characteristics. They obtained a breakdown voltage of 2070 V with a field plate of 2 μm and roughness of 0.6 nm, and a figure of merit FOM value of 1127 MW/cm^2^ was achieved [11]. In 2019, Sun [12] et al. investigated T-shaped anode structures by conducting a simulation and obtained a breakdown voltage of 1252 V and a specific on-state resistance of 0.32 mΩ·cm^2^. In 2019, Bu et al. prepared and compared SBD devices with and without a notched anode structure. They found that the notched anode had a superior overall performance with the same electrode spacing, and the device had a turn-on voltage of 0.75 V and a breakdown voltage of 462 V [13]. In 2020, Zhang et al. prepared a notched anode device using tungsten and obtained a low on-state voltage of 0.39 V [14]. In 2019, Zhang et al. prepared SBD devices using notched anodes and field plates and achieved a low turn-on voltage of 0.31 V and high reverse breakdown voltage of 2.65 KV with a figure of merit FOM of 2.65 GW/cm^2^.

This paper investigates the effect of electrode spacing, notch anode depth, and field plate length on device performance using Silvaco simulation software for recessed anode AlGaN/GaN SBD devices with field plate structure. The experimental flow results show that the recessed anode structure can reduce the turn-on voltage and reverse the leakage current of the device. The field plate structure can effectively improve the peak electric field at the anode edge of the device. At the same time, the forward characteristics and FOM can be further improved by optimizing the device structure through software simulation.

## 2. Device Design and Simulation Model

**Figure 1:**
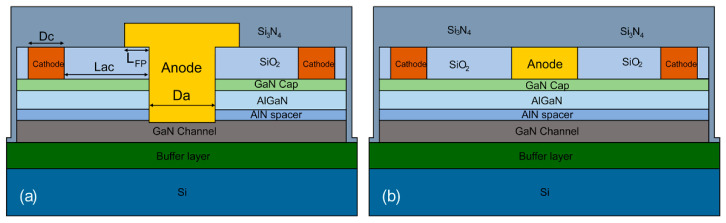
Schematic diagram of AlGaN/GaN SBD device structure: (**a**) field plate anode trench etched device; (**b**) field plate without anode trench etched device.

The AlGaN/GaN SBD device structure is shown in Figure 1. From bottom to top, the device consists of a Si substrate, 3 μm buffer layer, 500 nm GaN channel layer, 1 nm AlN spacer, 23 nm AlGaN barrier layer (Al content of 25%), 3 nm GaN cap layer, 300 nm SiO_2_, and 350 nm Si_3_N_4_. The anode width is denoted by Da, the cathode width is denoted by Dc, and the spacing between the anode and the cathode is denoted by Lac.

**Figure 2:**
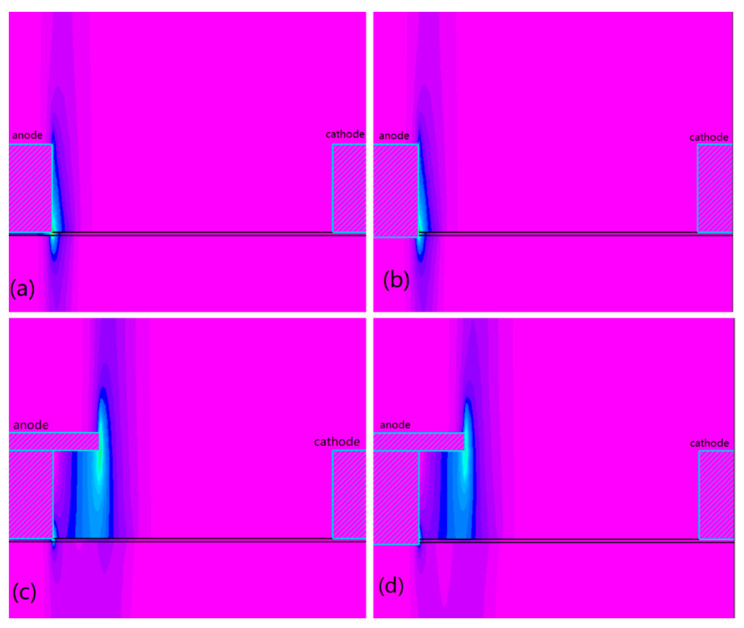
Simulation of device structure: (**a**) electric field breakdown of the device without field plate and no anode etching; (**b**) electric field breakdown of the device without field plate and anode etching; (**c**) electric field breakdown of the device with field plate and no anode etching; (**d**) breakdown of the device with field plate and anode etching.

In order to improve the performance of AlGaN/GaN SBD devices, the electrode spacing and field plate length of the AlGaN/GaN SBD devices were first simulated using the Silvaco TCAD software (Vancouver, BC, Canada). To simplify the simulation process, only half of the device structure was simulated, and the output characteristics of the device were consistent with those of the complete structure. The simulation of the device structure is shown in Figure 2, where the anode width Da = 100 μm and the cathode width Dc = 50 μm. Figure 2 shows the breakdown field diagrams with different device simulation designs, respectively.

The main physical models, which are used in the simulation, are as follows: field-dependent mobility (fldmob), Shockley–Read–Hall (SRH), Farahmand Modified Caughey Thomas (FMCT.N), Fermi–Dirac statistics (fermi), and domain-related mobility (print). The composite model (Auger) and collisional ionization model (selb) were used to simulate the breakdown.

The formula of collision ionization model is α_n_ = AN exp [−(BN/E)] and α_p_ = AP exp [−(BP/E)]. Here, α_n_ and α_p_ are the ionization coefficients of electrons and holes, and AN and BN are the lattice temperature correlation coefficients. The values of these physical parameters are defined in the model as AN = 7.03 × 10^5^ cm^−1^, AP = 6.71 × 10^5^ cm^−1^, BN = 1.231 × 10^6^ V/cm, and BP = 1.231 × 10^6^ V/cm.

The metal working function of the Schottky anode has been set to 5.15 eV, which is consistent with the work function of Ni used in the experimental flow sheet. The device parameters for the material used in the simulations are listed in Table 1.

**Figure 3:**
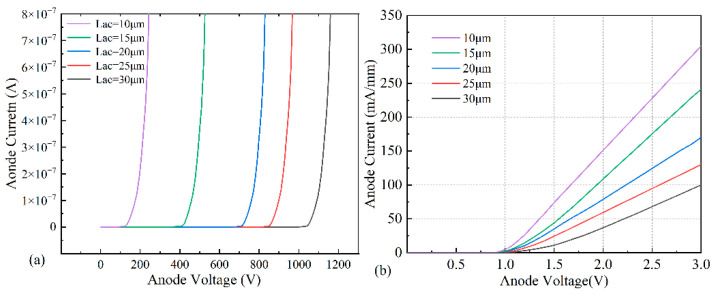
Simulation data of device: (**a**) breakdown voltage diagram with different electrode spacings; (**b**) forward I-V characteristic curves with different electrode spacings.

The breakdown characteristic curve of the device is shown in Figure 3a. As can be seen, the device’s breakdown voltage gradually increased with Lac, from 176 V at a pitch of 10 μm to 1092 V at a pitch of 30 μm. As the reverse voltage increased, the two-dimensional electron gas (2DEG) in the GaN channel layer was gradually depleted. The depletion zone gradually spread to the cathode with the increase in the reverse voltage until it was completely depleted. The increase in Lac extend the width of the depletion zone, such that V_RB_ = E_C_/W, where W is the width of the depletion zone, and E_C_ is the critical breakdown electric field; the magnitude of the breakdown voltage is proportional to the width of the depletion layer [16,17].

However, as the pitch increased, the series resistance in the device channel also increased, and the on-state resistance of the device increased. The variation of the forward I-V characteristics with Lac is shown in Figure 3b. The on-state resistance of the device increased from 6.49 Ω/mm at 10 μm to 15.5 Ω/mm at 30 μm. Considering the device performance and cost of the flow, the device performance is optimal when the device pitch is 20 μm.

**Figure 4:**
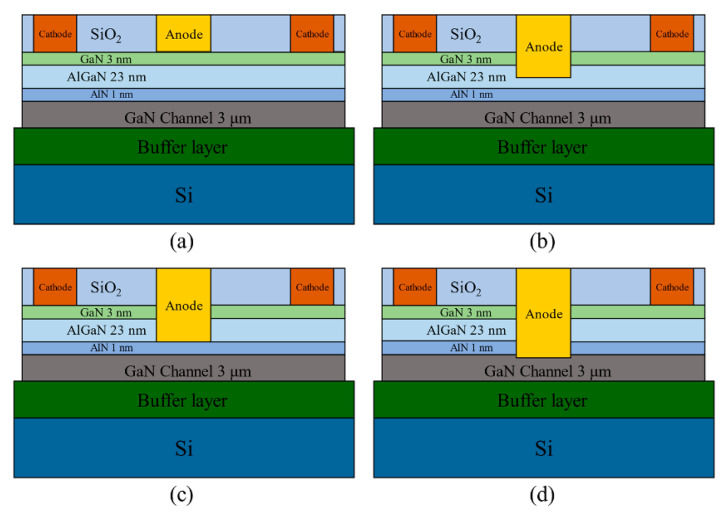
Schematic diagram of different etching depths. (**a**) no etch depth; (**b**) 13 nm etch depth; (**c**) 26 nm etch depth; (**d**) 30 nm etch depth.

The effects of different anode etching depths on the devices are individually simulated with an electrode spacing of 20 μm, as shown in Figure 4: (a) a no-etch device; (b) an etching depth of 13 nm such that the anode was partially inserted into the AlGaN layer; (c) an etching depth of 26 nm such that the anode is wholly inserted into the AlGaN layer; and (d) etching depth of 30 nm such that the bottom of the anode is in direct contact with the two-dimensional electron gas.

**Figure 5:**
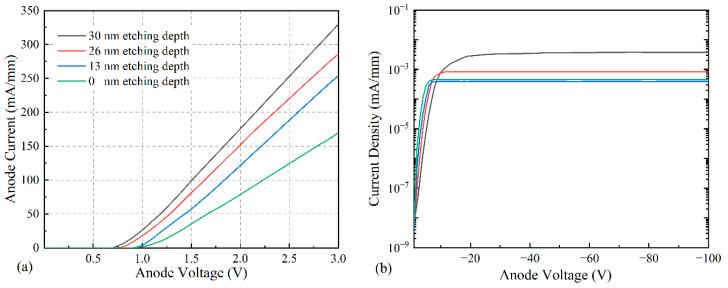
(**a**) Forward I-V characteristics at different etching depths; (**b**) leakage current diagram at different etching depths.

The results of the forward I-V characteristics of the simulated devices with different anode etching depths (Figure 4) are shown in Figure 5, where the corresponding turn-on voltages are 0.95 V, 0.86 V, 0.74 V, and 0.7 V for the etching depths of 0 nm, 13 nm, 26 nm, and 30 nm, respectively; the maximum current densities are 170 mA/mm, 254 mA/mm, 286 mA/mm, and 330 mA/mm for 3 V, respectively; the reverse leakage currents are 3.73 × 10^−3^ mA/mm, 8.41 × 10^−4^ mA/mm, 4.5 × 10^−4^ mA/mm, and 3.96 × 10^−4^ mA/mm, respectively. According to Figure 5, the device has the lowest turn-on voltage, highest forward current density, and lowest leakage current when the etching depth is 30 nm.

When the AlGaN/GaN SBD device is in reverse bias, according to the edge electric field concentration effect, the distribution of the electric field at the edge of the electrode is not uniform. In addition, the electric field distribution becomes more concentrated as the distance to the electrode edge decreases, and the depletion layer at the edge of the Schottky electrode will reach the critical breakdown electric field early, causing the breakdown of the device. By adding the structure of the field plate, the peak electric field at the anode edge of the device can be weakened, preventing the premature breakdown of the device due to the concentration of the electric field [18].

**Figure 6:**
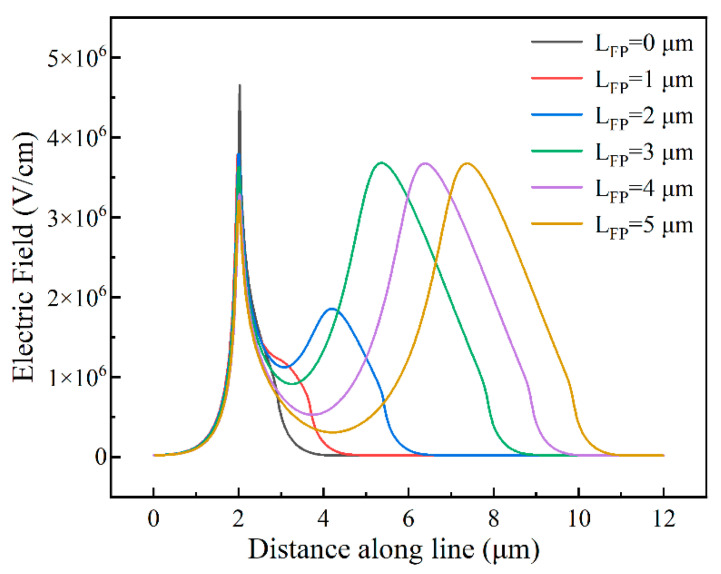
Simulation of electric field intensity distribution for different field plate lengths.

Therefore, we simulated devices with different field plate lengths with a device spacing of 20 μm to investigate the effect of the field plate on the reverse breakdown electric field distribution. The electric field of the simulated device (Figure 2) was cut and measured, and the electric field distribution is shown in Figure 6.

According to Figure 6, at L_FP_ = 0 μm, the electric field distribution has only one peak at the edge of the Schottky electrode. When the field plate was added, the electric field peak split into two peaks, one at the Schottky edge and the other at the field plate edge. At the same reverse voltage, the device with no field plate only bore the voltage at the Schottky electrode edge. The peak electric field of the Schottky electrode with the field plate was reduced, part of the electric field was shared with the field plate, and the corresponding reverse breakdown voltage of the device increased.

**Figure 7:**
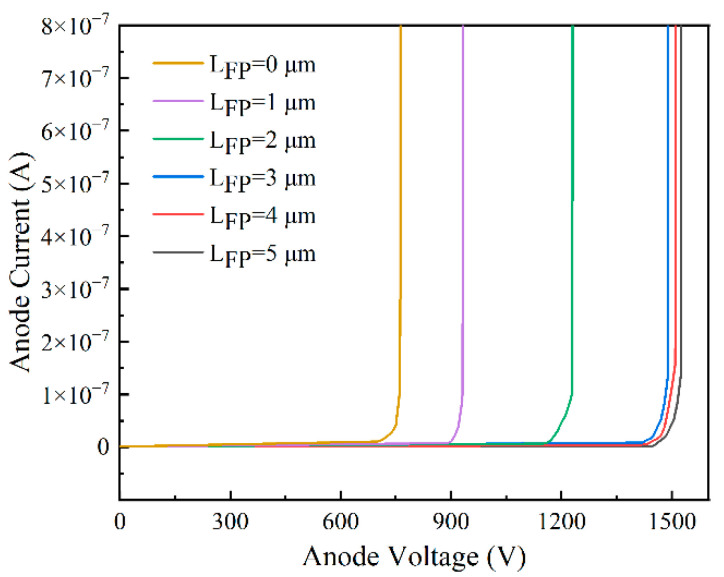
Breakdown voltage diagram for different simulated field plate lengths.

Figure 7 shows that the increased length of the field plate led to an increased device breakdown voltage. After the field plate length exceeded 3 μm, the field plate provided a small enhancement for the breakdown voltage, from 1489 V at L_FP_ = 3 μm to 1513 V at L_FP_ = 5 μm. Then, according to the electric field distribution diagram in Figure 6, when the field plate length was greater than 3 μm, the second peak of the device, that is, the electric field peak at the field plate edge no more increased, the electric field sharing of the field plate for the device has reached the maximum, and the breakdown voltage tended to be constant. Therefore, a field plate of 3 μm is the optimal length of the field plate for the device.

From the viewpoint of device structure improvement through software simulation, the simulation above compared the forward and reverse characteristics of different electrode spacings, and the best overall device performance was achieved at Lac = 20 μm. By simulating the influence of etching depth on the device’s forward performance, the device forward current reached the maximum value when the etching depth was 30 nm, and the anode metal was directly [19] in a two-dimensional electron (2DEG) contact. The effect of different field plate lengths on the breakdown characteristics of the device was also simulated. The breakdown voltage of the device increased significantly with increased field plate length, but after the length of the field plate exceeded 3 μm, the field plate structure has little effect on increasing the breakdown voltage, and the field plate’s contribution to the peak electric field was maximized. By sharing the peak electric field [20,21] at the anodic edge of the device, the field plate structure weakens the edge electric field concentration effect, thus increasing the breakdown voltage of the device.

## 3. Experiments and Tests

**Figure 8:**
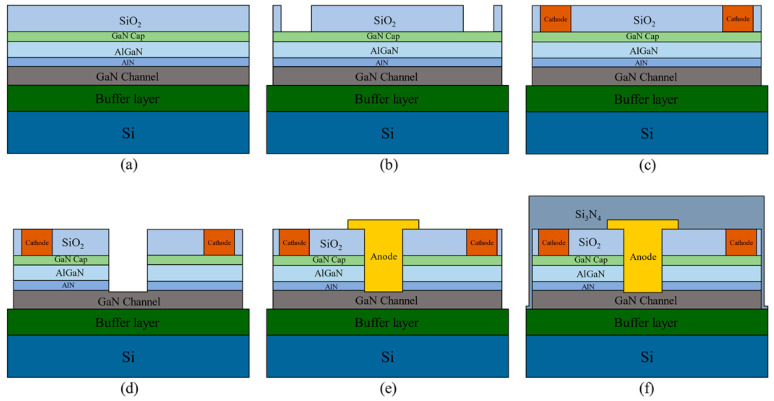
Flow chart of device preparation. (**a**) SiO_2_ surface deposition passivation; (**b**) bench isolation etching and ohmic trench etching; (**c**) ohmic metal deposition; (**d**) Schottky anode trench etching; (**e**) Schottky metal deposition; and (**f**) Si_3_N_4_ surface deposition passivation.

The epitaxial wafer structure used in the simulation is identical to the simulated structure. From top to bottom, they are composed of a 3 nm GaN cap layer, 23 nm AlGaN layer, 1 nm AlN transition layer, 500 nm undoped GaN, 3 μm GaN buffer layer, and Si substrate. The manufacturing procedure of the SBD is illustrated in Figure 8.

The Ohmic metal material used in the experiments is Ti/Al/Ni/Au. The Rapid Thermal Anneal (RTA) conditions are N_2_ atmosphere, an annealing temperature of 850 °C, and a time of 30 s. The Schottky metal material is Ni/Au, and the annealing conditions are a N_2_ atmosphere of 500 °C and an annealing time of 10 min. In this study, AlGaN/GaN SBD devices with different field plate structures and etching depths at the electrode spacing Lac = 20 μm are experimentally prepared.

**Figure 9:**
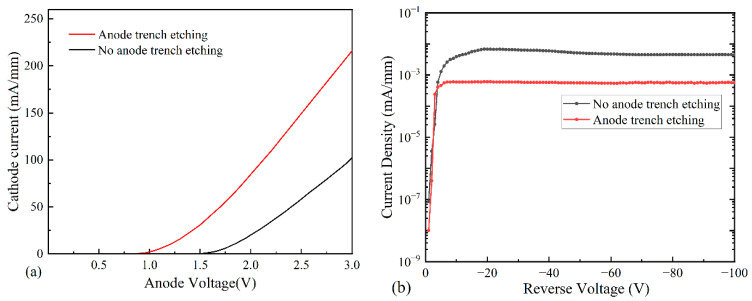
Comparison of unetched device and etched device: (**a**) forward current comparison; (**b**) reverse leakage current comparison.

Figure 9 shows the comparison of the forward characteristics and leakage currents for the anodic trench etched and unetched devices with turn-on voltages of 0.75 V and 1.5 V, respectively, and a turn-on voltage reduction of 0.75 V. The forward current densities were 216 mA/mm and 98 mA/mm; the differential on-resistance is 8.3 Ω·mm and 14.7 Ω·mm, at 3 V, respectively. Additionally, the corresponding leakage currents at the bias voltage of −100 V are 5.7 × 10^−4^ mA/mm and 4.5 × 10^−3^ mA/mm, respectively, and the reverse leakage is reduced by 87.3%. The anode notch etching treatment allows the anode metal to directly contact the two-dimensional electron gas, which reduces the electron movement distance and thus reduces the turn-on voltage and reverse leakage current.

**Figure 10:**
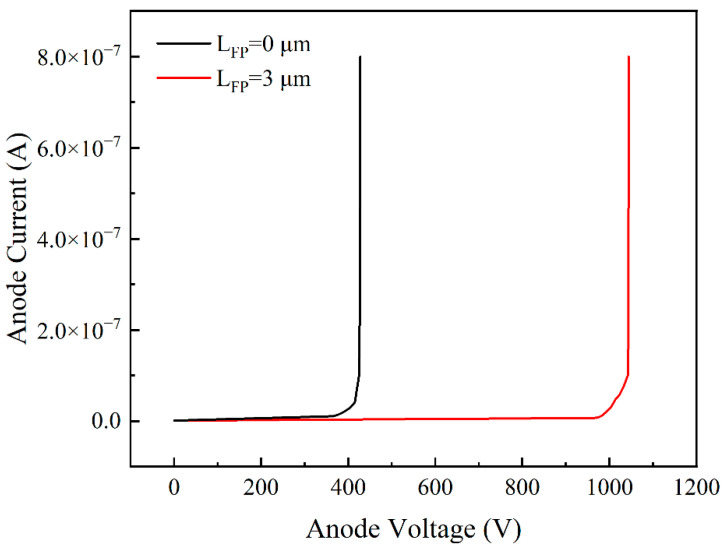
Breakdown voltage diagram for different field plates.

Figure 10 shows the breakdown voltage plots for devices with no field plate and devices with a 3 μm field plate when Lac was 20 μm, and the etching depth was 30 nm. When the structure of the field plate is 3 μm, the devices had an increased breakdown voltage from 427 V with no field plate to 1043 V. Therefore, the breakdown voltage significantly improved.

As an essential index for assessing the goodness of the device, FOM [22] can be calculated by the formula below:

FOM = V_BV_^2^/Ron.sp

where V_BV_ is the breakdown voltage, and Ron.sp is the specific on-resistance of the device. According to the equation, the FOM value of the device can be increased by increasing the breakdown voltage and specific on-resistance. In summary, the device achieves the maximum FOM of 572.6 MW/cm^2^ with an electrode spacing of 20 μm, 3 μm field plate, and an etching depth of 30 nm. The data obtained by the experiment are presented in Table 2.

## 4. Conclusions

In this paper, the best device performance is obtained at 20 μm electrode pitch, 30 nm etch depth, and 3 μm field plate length by simulation using silvaco-TCAD software, and different types of devices are prepared by experiment. The experimental results are consistent with the simulation. The recessed anode treatment can improve the forward characteristics of the device and reduce the turn-on voltage and reverse leakage current. The field plate structure can increase the breakdown voltage of the device.

The recessed anode treatment and field plate structure produce an AlGaN/GaN SBD device with a breakdown voltage of 1043 V, an intrinsic on-resistance of 1.9 mΩ·cm^2^, and a FOM value of 572.6 MW/cm^2^. The break-down voltage of the 3 μm FP is about 2.4 times that of the no FP, 0.75 V reduction in turn-on voltage, and 87.3% reduction in reverse leakage current compared to devices with no recessed anode. The device’s overall performance has been improved. The results show promising results for the recessed anode treatment and the field plate structure. Simulation prior to experimental preparation can save time and cost.

## Figures and Tables

**Table 1:** Parameters for simulation [15].

Parameters	GaN	AlGaN
Eg300 (eV)	3.4	3.96
Align	0.8	0.8
Permittivity	9.5	9.5
Electron Saturation Velocity (Vsat)	2 × 10^7^ cm/s	1.1 × 10^7^ cm/s
Electronic Low Field Mobility (Mun)	900 cm^2^/Vs	600 cm^2^/Vs
Hole Low Field Mobility (Mup)	10 cm^2^/Vs	10 cm^2^/Vs
300K conduction band state density (Nc 300)	2.07 × 10^18^ cm^3^	2.07 × 10^18^ cm^3^
300K valance band state density (Nv 300)	1.16 × 10^18^ cm^3^	1.16 × 10^18^ cm^3^

**Table 2:** Device test parameters.

		No Recesed Anode		30 nm Recessed Anode	
		FP = 0 μm	FP = 3 μm	FP = 0 μm	FP = 3 μm
Von (V)		1.5	1.5	0.75	0.75
Ron (Ω·mm)		14.7	14.6	8.4	8.3
Ron.sp (mΩ·cm^2^)		3.38	3.35	1.93	1.9
V_BV_ (V)		427	1043	427	1043
FOM (MW/cm^2^)		53.9	324.7	94.5	572.6

## Data Availability

No applicable.
